# Luteolin Alleviates Epithelial-Mesenchymal Transformation Induced by Oxidative Injury in ARPE-19 Cell *via* Nrf2 and AKT/GSK-3*β* Pathway

**DOI:** 10.1155/2022/2265725

**Published:** 2022-02-14

**Authors:** Lan Chen, Yanqing Zhu, Jie Zhou, Rui Wu, Ning Yang, Qinbin Bao, Xinrong Xu

**Affiliations:** Department of Ophthalmology, Jiangsu Province Hospital of Chinese Medicine (Affiliated Hospital of Nanjing University of Chinese Medicine), Nanjing 210029, China

## Abstract

Oxidative stress plays a critical role in age-related macular degeneration (AMD), and epithelial-mesenchymal transition (EMT) is involved in this process. The aim of this study was to investigate the protective effects of luteolin, a natural flavonoid with strong antioxidant activity, on H_2_O_2_-induced EMT in ARPE-19 cells. ARPE-19 cells were incubated with H_2_O_2_ at 200 *μΜ* to induce oxidative stress-associated injury. Cell viability assay showed that luteolin at 20 and 40 *μ*M significantly promoted cell survival in H_2_O_2_-treated ARPE-19 cells. Luteolin also markedly protected ARPE-19 cells from H_2_O_2_-induced apoptosis. Cell migration assay presented that luteolin significantly reduced H_2_O_2_-induced migration in APRE-19 cells. EMT in ARPE-19 cells was detected by western blotting and immunofluorescence. The results showed that H_2_O_2_ significantly upregulated the expression of *α*-SMA and vimentin and downregulated the expression of ZO-1 and E-cadherin, while cells pretreated with luteolin showed a reversal. Meanwhile, the assessment of effects of luteolin on the Nrf2 pathway indicated that luteolin promoted Nrf2 nuclear translocation and upregulated the expressions of HO-1 and NQO-1. In addition, luteolin significantly increased the activities of SOD and GSH-PX and decreased intracellular levels of ROS and MDA in H_2_O_2_-treated ARPE-19 cells. Meanwhile, we observed that the expression of TGF-*β*2, p-AKT, and p-GSK-3*β* was upregulated in H_2_O_2_-treated ARPE-19 cells and downregulated in luteolin-treated cells, revealing that luteolin inhibited the activation of the AKT/GSK-3*β* pathway. However, these effects of luteolin were all annulled by transfecting ARPE-19 cells with Nrf2 siRNA. Our current data collectively indicated that inhibition of luteolin on EMT was induced by oxidative injury in ARPE-19 cell through the Nrf2 and AKT/GSK-3*β* pathway, suggesting that luteolin could be a potential drug for the treatment of dry AMD.

## 1. Introduction

Age-related macular degeneration (AMD) is the leading cause of vision loss in the elderly [1]. From current knowledge, AMD is a multifactorial degenerative disease of the retina. In addition to aging, susceptibility genes, environmental factors, chronic localized inflammation, and choroidal vascular dysfunction all play important roles in the onset and progression of AMD [2]. Retinal pigment epithelium (RPE) is a monolayer cell located between photoreceptors and Bruch's membrane, characterized by cell-cell adhesion and apical-basal polarity arrangement. RPE transports nutrients from the choroidal capillary layer to the photoreceptors and phagocyte and digests photoreceptor outer segments, which is critical for keeping the photoreceptor function [3]. Abnormal distribution and dysfunction of RPE cells lead to retinal degeneration and eventually to loss of vision.

Epithelial-mesenchymal transition (EMT) is a physiological process necessary for normal embryonic development. There are three types of EMT, of which type 2 EMT is associated with tissue regeneration and wound healing, but its abnormal activation enhances pathological tissue fibrosis [4]. Tissue damage, oxidative stress, and inflammation are important factors to activate EMT. Many literatures support the role of EMT in AMD. In dry AMD, RPE loses its characteristic shape and phenotype, showing migration in the transition zone between atrophy and normal retina [5]. Further histological examination of these “degenerating” cells showed that they may not be dying but reversibly transformed into mesenchymal cells through EMT [6]. Multiple extracellular ligands are involved in the initiation and progression of the EMT process. Transforming growth factor-beta (TGF-*β*) is considered to be the master regulator of the process [7]. Reactive oxygen species (ROS) can directly activate TGF-*β*1 and initiate the EMT process through the PI3K/AKT and MAPK pathway, which means that the control of oxidative stress has the potential to regulate EMT [8]. The inhibition of the mTORC1-NOX4 pathway by an activator of AMP-dependent protein kinase, or a NOX1/4 inhibitor, decreased ROS generation, prevented stress fiber formation, attenuated EMT, and improved the regularity of the RPE structure in vitro and in vivo [9]. The Nrf2 pathway is the most important antioxidant stress defense pathway in the human body and plays an important role in the pathogenesis of AMD [10, 11]. ROS production is increased under oxidative stress, which activates the Nrf2 pathway to enhance the ability of cells to clear ROS, maintain the balance of intracellular redox state, and reduce oxidative damage. Therefore, activation of the Nrf2 pathway is considered to be a potential therapeutic strategy for AMD [12, 13]. Some studies have confirmed that the Nrf2 pathway is involved in the regulation of EMT [14, 15]. It is thus expected that the interaction between the Nrf2 pathway and EMT, two important events under oxidative stress, may significantly expand the role of antioxidants in the treatment of AMD.

Luteolin is a natural flavonoid found in many plants, such as carrots, broccoli, perilla leaves, and celery. Luteolin has a variety of biological effects, such as anti-inflammatory, antioxidant, and antitumor [16–18]. Recent studies have found that many flavonoids can inhibit EMT. Tangeretin inhibits podocyte damage and fibrosis by blocking glucose-induced oxidative stress and hypoxia-induced podocyte EMT [19]. Galatechin-3-gallate attenuates TGF-*β*1-induced renal tubular cell EMT through GSK-3*β*/*β*-catenin/snail1 and Nrf2 pathways [20]. It has been confirmed that luteolin has a significant inhibition on EMT in tumor cells [21, 22], but it is unclear whether it also inhibits EMT in RPE cells. In this study, we used ARPE-19 cells, a commercial human RPE cell line widely used in AMD research, to assess the effect of luteolin on H_2_O_2_-induced EMT, focusing on the involvement of the Nrf2 and AKT/GSK-3*β* pathway in EMT.

## 2. Materials and Methods

### 2.1. Regents and Antibodies

Hydrogen peroxide (H_2_O_2_) and luteolin were purchased from Sigma-Aldrich (Saint Louis, MO, USA). The primary antibodies against E-cadherin, *α*-SMA, GSK-3*β*, p-GSK-3*β*, AKT, and p-AKT were from Cell Signaling Technology (Andover, MA, USA). ZO-1, vimentin, Nrf2, HO-1, NQO-1, TGF-*β*2, GAPDH, Lamin B, and the secondary antibody Goat Anti-Rabbit IgG/HRP were from Abcam (Cambridge, UK).

### 2.2. Cell Culture

ARPE-19 was obtained from the American Type Culture Collection (USA). ARPE-19 cells were cultured in Dulbecco's modified eagle medium (DMEM) supplemented with 10% fetal bovine serum (FBS) and 1% penicillin/streptomycin in humidified air at 37°C with 5% CO_2_. After reaching confluence, cells were detached from culture flasks using Trypsin-EDTA, washed, and resuspended in a complete medium.

### 2.3. Cell Transfection with Nrf2 siRNA

For siRNA transfection, ARPE-19 cells were subjected to transient transfection with Nrf2-negative control siRNA or Nrf2-siRNA (Shanghai GenePharma Co. Ltd., Shanghai, China) using the siRNA transfection reagent Lipofectamine™ 2000 reagent (Invitrogen, USA) following the manufacturer's protocol.

### 2.4. Cell Proliferation Assay

ARPE-19 cells were seeded in 96-well plates (1 × 10^4^/well) and cultured in DMEM with 10% FBS for 24 h. Cells were treated with vehicle (DMSO) and luteolin at concentrations of 10, 20, and 40 *μ*M for 24 h and then additionally treated with H_2_O_2_ (200 *μ*M) for 24 h. Then, the medium was replaced with 100 *μ*L phosphate-buffered saline (PBS) containing 0.5 mg/mL MTT incubating at 37°C for 4 h. MTT solution was then removed, and 100 mL DMSO was added to each well, followed by mixing for 10 min, and the OD value was measured at 490 nm using a SpectraMax™ microplate spectrophotometer (Molecular Devices, Sunnyvale, CA, USA).

### 2.5. Cell Migration Assay

A 24-well modified Boyden chamber (Corning, NY, USA) was performed in migration assay. ARPE-19 cells (1 × 10^5^/mL) from the indicated groups were resuspended in the upper chambers with 200 *μ*L serum-free MEM. The bottom chambers were filled with 500 *μ*L of DMEM containing 10% FBS. The chambers were incubated at 37°C and 5% CO_2_ for 24 h. Nonmigrated cells on the top surface of the filter were wiped away using cotton swabs. The cells that migrated through the filter were fixed with 4% paraformaldehyde for 10 min and stained with 1% crystal violet in methanol. After washing twice with PBS, the cell number of the bottom side was counted and plotted as the mean number of cells migrated in six nonoverlapping fields by three investigators under a microscope (Olympus IX70, Tokyo, Japan).

### 2.6. Assay of Apoptosis

Assay of apoptosis was performed as previously described [23]. Briefly, ARPE-19 cells were treated with vehicle (DMSO) and luteolin at indicated concentrations for 24 h and then additionally treated with H_2_O_2_ (200 *μ*M) for 24 h. Apoptotic rates were determined by flow cytometry using Annexin V-FITC apoptosis assay kits (Nanjing KeyGen Biotech Co., Ltd., Nanjing, China) according to the protocol. The apoptotic rate of ARPE-19 cells was determined by flow cytometry (FACSCalibur, Becton, Dickinson and Company, NJ, USA). The data was analyzed using the software CellQuest. All these experiments were performed in triplicate.

### 2.7. Determination of Activities of Antioxidases

ARPE-19 cells were treated with vehicle (DMSO) and luteolin at indicated concentrations for 24 h and then additionally treated with H_2_O_2_ (200 *μ*M) for 24 h. Cells were broken up by ultrasound treatment and subjected to centrifugation. Each supernatant of 100 *μ*L was used to detect the activities of superoxide dismutase (SOD) and GSH-PX using their enzyme-linked immunosorbent assay kits (Nanjing Jiancheng Bioengineering Institute, Nanjing, China) according to the protocols. Experiments were performed in triplicate.

### 2.8. Assay of Intracellular ROS and MDA

The fluorescent probe DCFH-DA was used to measure the intracellular ROS production. Cells were treated with 10 *μ*M DCFH-DA for 20 min at 37°C. After incubating with DMSO and luteolin at indicated concentrations for 24 h and then additionally treating with H_2_O_2_ (200 *μ*M) for 24 h, the fluorescence was determined by flow cytometry (FACSCalibur, Becton, Dickinson and Company, NJ, USA) at 488 nm wavelength. MDA levels were detected by the thiobarbituric acid (TBA) method. ARPE-19 cells were broken up by ultrasound treatment and subjected to centrifugation. Levels of MDA in supernatants were measured using a kit (Nanjing Jiancheng Bioengineering Institute, Nanjing, China). Experiments were performed in triplicate.

### 2.9. Immunofluorescence Assay

ARPE-19 cells were seeded on 8-well cell culture plates. After treatment with 40 *μ*M of luteolin or DMSO as control, the cells were then washed with PBS, fixed with 4% paraformaldehyde for 15 min, permeabilized with 0.5% Triton X-100 for 5 min at room temperature, and blocked with 1% bovine serum albumin in PBS for 1 h. The fixed cells were incubated with primary antibodies against ZO-1 and vimentin at 4°C overnight, then washed with PBS, and incubated with the appropriate secondary antibody (Alexa Fluor® 488 goat anti-rabbit immunoglobulin IgG, Invitrogen Corporation) followed by DNA staining with DAPI for 5 min at room temperature. Then, the cell culture plates were mounted on glass slides and observed under a laser confocal microscope (Zeiss LSM880, Carl Zeiss, Oberkochen, Germany) equipped with ZEN image processing software.

### 2.10. Western Blotting

After incubating with DMSO and luteolin at indicated concentrations for 24 h and then additionally treating with H_2_O_2_ (200 *μ*M) for 24 h, the total protein of ARPE-19 cells was extracted with radioimmunoprecipitation assay buffer containing 1 mM PMSF and quantified using the BCA protein assay kit (Nanjing Jiancheng Bioengineering Institute, Nanjing, China). Equal amounts of protein (20 *μ*g) were separated by 10% SDS-PAGE and then transferred to a PVDF membrane (Bio-Rad Laboratories, Hercules, CA, USA), which was blocked with 5% skimmed milk in TBST at room temperature for 2 h. Afterward, the membrane was incubated with antibodies against Nrf2 (dilution, 1 : 1000), snail (dilution, 1 : 1000), HO-1 (dilution, 1 : 1000), NQO-1 (dilution, 1 : 1000), *α*-SMA (dilution, 1 : 500), E-cadherin (dilution, 1 : 1000), TGF-*β*2 (dilution, 1 : 1000), GSK-3*β* (dilution, 1 : 1000), p-GSK-3*β* (dilution, 1 : 1000), AKT (dilution, 1 : 1000), p-AKT (dilution, 1 : 500), Lamin B (dilution, 1 : 1000), and GAPDH (dilution, 1 : 1000) overnight at 4°C. Lamin B (for nuclear protein) or GAPDH (for total proteins) was used as an internal control. The reacted membranes were incubated with HRP-conjugated secondary antibody for 2 h at room temperature. An enhanced chemiluminescence reagent kit (EMD Millipore Corporation) was used to detect the immune complexes. The blots were scanned, and the band intensity was analyzed using ImageJ software (NIH, version 1.48).

### 2.11. Statistical Analysis

All experiments were performed at least three times. Data were presented as mean ± SD, and results were analyzed using GraphPad Prism 7.0 software (GraphPad Software, La Jolla, CA, USA). The significance of the difference was determined by one-way ANOVA with the post hoc Dunnett test. *P* < 0.05 was considered to be statistically significant.

## 3. Results

### 3.1. Luteolin Protected Cells against Oxidative Injury

**Figure 1:**
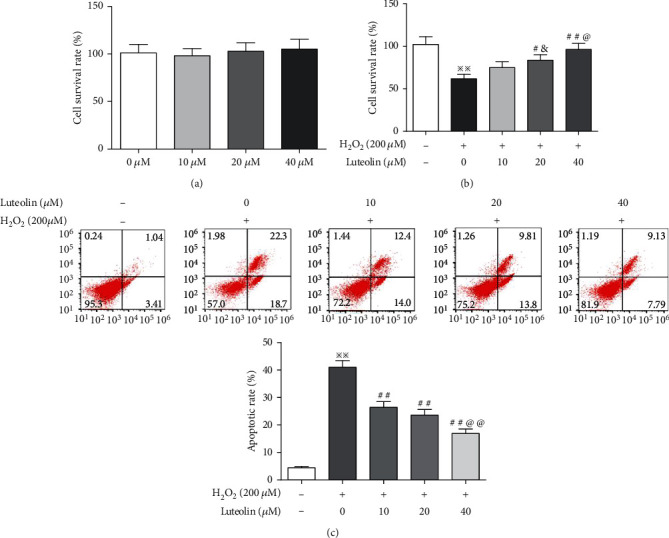
Luteolin protected ARPE-19 cell against H_2_O_2_-induced oxidative Injury. (a) MTT assay for evaluating the effect of luteolin on cell proliferation. (b) MTT assay for evaluating the protective effects of luteolin on H_2_O_2_-treated cells. (c) Flow cytometry for evaluating the effect of luteolin on apoptosis in H_2_O_2_-treated cells. Data are expressed as mean ± SD (*n* = 3). ^※※^*P* < 0.01 versus normal control; ^#^*P* < 0.05 and ^##^*P* < 0.01 versus H_2_O_2_; ^&^*P* < 0.05 versus luteolin 10 *μ*M; ^@^*P* < 0.05 and ^@@^*P* < 0.01 versus luteolin 20 *μ*M.

The potential cytotoxicity of varying concentrations of luteolin on ARPE-19 cells was determined by MTT. As shown in Figure 1(a), luteolin at the doses used (10, 20, and 40 mM) did not apparently affect ARPE-19 cell viability. Thus, we used these doses of luteolin in this experiment. H_2_O_2_, a widely used oxidizing agent, was used to induce oxidative damage in APRE-19. Our previous results showed that the viability of ARPE-19 cells treated with H_2_O_2_ at 200 *μ*M decreased to 63% [24]. Therefore, 200 *μ*M H_2_O_2_ was used for subsequent experiments. Then, we evaluated the effect of luteolin on the survival rate of H_2_O_2_-treated ARPE-19 cells. As shown in Figure 1(b), luteolin increased cell survival rate in a dose-dependent manner. Next, apoptosis was examined using flow cytometry. As shown in Figure 1(c), ARPE-19 cells exposed to H_2_O_2_ had significant apoptotic rate compared to control, but luteolin significantly reduced apoptosis. These data collectively indicated that luteolin effectively protected against cell death in ARPE-19 cells treated with H_2_O_2_.

### 3.2. Luteolin Suppressed the Cell Migration

**Figure 2:**
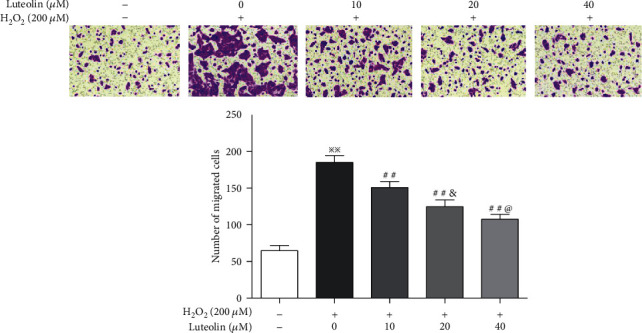
Luteolin suppressed the cell migration in H_2_O_2_-treated ARPE-19 cells. APRE-19 cells were treated with H_2_O_2_ and luteolin at the indicated concentrations. Transwell migration for assessment of the effect of luteolin on cell migration. Data are expressed as mean ± SD (*n* = 3). Scale bar = 200 *μ*m. ^※※^*P* < 0.01 versus normal control; ^##^*P* < 0.01 versus H_2_O_2_; ^&^*P* < 0.05 versus luteolin 10 *μ*M; ^@^*P* < 0.05 versus luteolin 20 *μ*M.

RPE cell migration is an important step during AMD progress. Transwell migration assays showed that the number of cells crossing the membrane was significantly higher in H_2_O_2_-treated cells than in normal cells; luteolin significantly reduced H_2_O_2_-induced migration in APRE-19 cells in a dose-dependent manner (Figure 2).

### 3.3. Luteolin Attenuated the H_2_O_2_-Induced EMT

**Figure 3:**
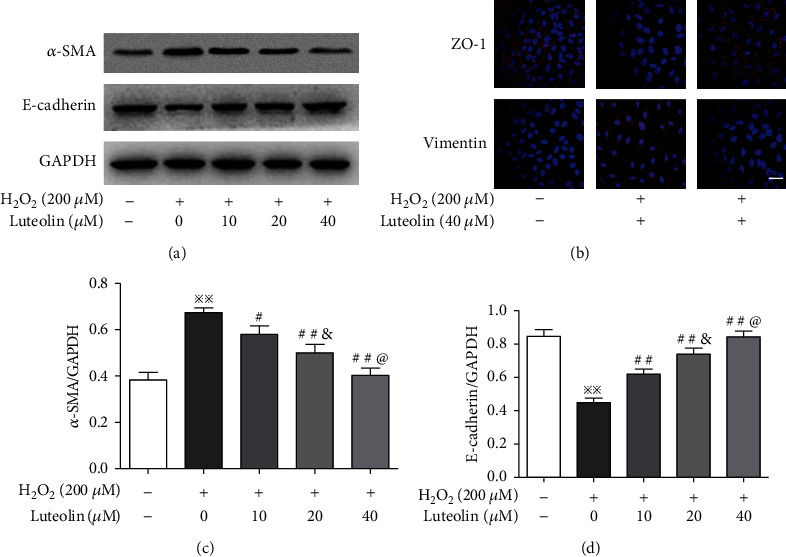
Luteolin attenuated the H_2_O_2_-induced EMT. APRE-19 cells were treated with H_2_O_2_ and luteolin at the indicated concentrations. (a, c, d) Western blot analyses of protein abundance of *α*-SMA and E-cadherin with quantification. Data are expressed as mean ± SD (*n* = 3). ^※※^*P* < 0.01 versus normal control; ^#^*P* < 0.05 and ^##^*P* < 0.01 versus H_2_O_2_; ^&^*P* < 0.05 versus luteolin 10 *μ*M; ^@^*P* < 0.05 versus luteolin 20 *μ*M. (b) Immunofluorescence for examining expression levels and localizations of ZO-1 and vimentin. Scale bar = 200 *μ*m.

EMT in ARPE-19 cells was detected by western blotting and immunofluorescence to measure expression levels of both epithelial and mesenchymal markers. Western blotting revealed significant upregulation of *α*-SMA and downregulation of E-cadherin in ARPE-19 cells treated with H_2_O_2_ (Figures 3(a), 3(c), and 3(d)). Similarly, immunofluorescence test demonstrated decreased expression of ZO-1 and increased expression of vimentin (Figure 3(b)). These results indicated that under oxidative stress, ARPE-19 cells altered their epithelial phenotypes. However, the expressions of these markers were reversed in ARPE-19 cells pretreated with luteolin. Our results indicated that luteolin inhibited EMT in ARPE-19 cells induced by H_2_O_2_.

### 3.4. Luteolin Promoted Nrf2 Nuclear Translocation and Increased Antioxidant Enzyme Expressions

**Figure 4:**
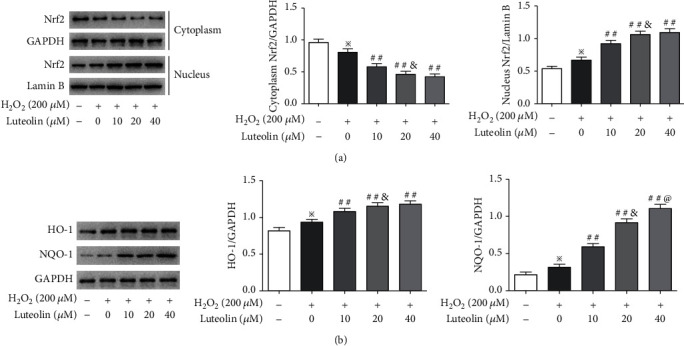
Luteolin promoted Nrf2 nuclear translocation and upregulated antioxidant enzyme expressions. APRE-19 cells were treated with H_2_O_2_ and luteolin at the indicated concentrations. (a) Western blotting to evaluate levels of Nrf2 in the nucleus and cytoplasm, respectively, and (b) the expression of HO-1 and NQO-1. Data are expressed as mean ± SD (*n* = 3). ^※^*P* < 0.05 versus normal control; ^##^*P* < 0.01 versus H_2_O_2_; ^&^*P* < 0.05 versus luteolin 10 *μ*M; ^@^*P* < 0.05 versus luteolin 20 *μ*M.

We assessed the effects of luteolin on the Nrf2 pathway. As shown in Figure 4(a), the level of cytoplasmic Nrf2 was decreased and the level of nuclear Nrf2 was increased in ARPE-19 cells treated with H_2_O_2_. Luteolin reduced the level of cytoplasmic Nrf2 and increased the level of nuclear Nrf2 in a dose-effect manner, indicating that luteolin promoted Nrf2 nuclear translocation. Meanwhile, we examined the expression of HO-1 and NQO-1, target proteins of the Nrf2 pathway. As shown in Figure 4(b), the expressions of HO-1 and NQO-1 were increased in ARPE-19 cells treated with H_2_O_2_ and significantly upregulated when pretreated with luteolin.

### 3.5. Luteolin Exhibits Potent Antioxidative Activities in ARPE-19 Cells Treated with H_2_O_2_

**Figure 5:**
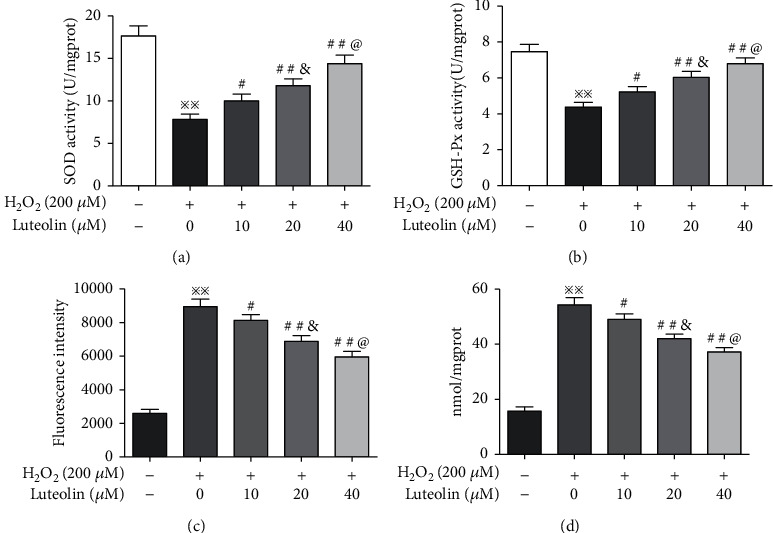
Luteolin protects against H_2_O_2_-induced oxidative injury in ARPE-19 cells. APRE-19 cells were treated with H_2_O_2_ and luteolin at the indicated concentrations. (a, b) The activities of SOD and GSH-PX by enzyme-linked immunosorbent assay. (c) The level of ROS was measured with the DCFH-DA method. (d) The level of MDA was detected with the TBA method. Data are expressed as mean ± SD (*n* = 3). ^※※^*P* < 0.01 versus normal control; ^#^*P* < 0.05, ^##^*P* < 0.01 versus H_2_O_2_; ^&^*P* < 0.05 versus luteolin 10 *μ*M; ^@^*P* < 0.05 versus luteolin 20 *μ*M.

We next examined the effects of luteolin on enzyme activities of SOD and GSH-PX. The results demonstrated that the activities of SOD and GSH-PX were significantly reduced in H_2_O_2_-treated ARPE-19 cells, but luteolin significantly restored the activities of these enzymes (Figures 5(a) and 5(b)). The exposure of the retina to ROS is thought to be a crucial factor in the development of AMD. ROS causes lipid peroxidation of the biomembrane, which leads to the production of a large amount of MDA [25]. Therefore, we measured the levels of ROS and MDA in ARPE-19 cells treated with H_2_O_2_. The results showed that the levels of ROS and MDA were significantly elevated in ARPE-19 cells treated with H_2_O_2_ and reduced in ARPE-19 cells pretreated with luteolin (Figures 5(c) and 5(d)).

### 3.6. Luteolin Inhibited the Activation of the AKT/GSK-3*β* Signaling Pathway

**Figure 6:**
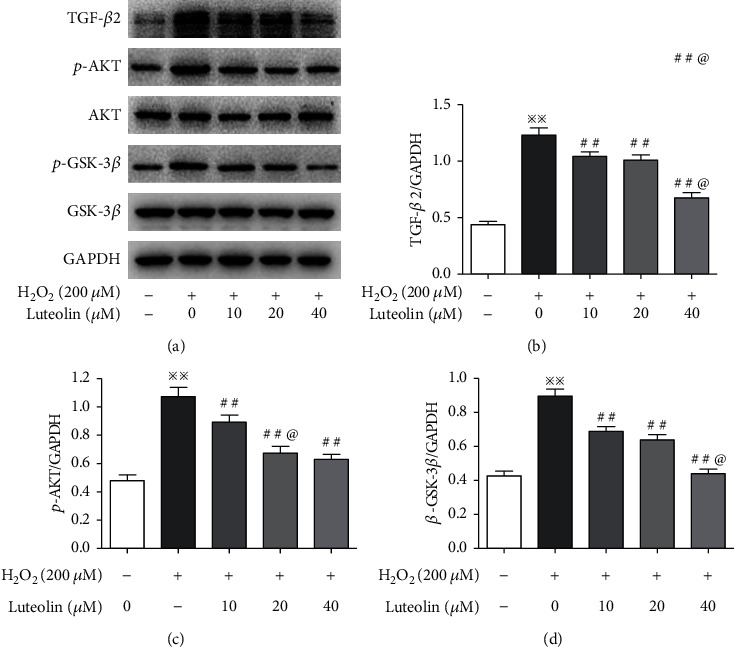
Luteolin inhibited the activation of the AKT/GSK-3*β* signaling pathway. APRE-19 cells were treated with H_2_O_2_ and luteolin at the indicated concentrations. (a–d) Western blotting to evaluate the expression of TGF-*β*2, AKT, p-AKT, GSK-3*β*, and p-GSK-3*β*. Data are expressed as mean ± SD (*n* = 3). ^※※^*P* < 0.01 versus normal control; ^##^*P* < 0.01 versus H_2_O_2_; *P* < 0.05 versus luteolin 20 *μ*M.

The signaling for EMT mediated by TGF-*β* is directly activated by ROS. The presence of ROS allows multiple growth factors to more easily dock with tyrosine kinase receptors, which leads to GSK-3*β* inactivation via PI3K/Akt pathways. Subsequent activation of *β*-catenin triggers EMT [26]. Therefore, we first detected the expression of TGF-*β*2. Compared to the normal cells, the expression of TGF-*β*2 was significantly increased in H_2_O_2_-treated cells and decreased in luteolin-treated cells. Then AKT, p-AKT, GSK-3*β*, and p-GSK-3*β* were evaluated. We found that p-AKT and p-GSK-3*β* significantly increased in H_2_O_2_-treated cells and decreased in luteolin-treated cells (Figure 6). Taken together, these results indicated that luteolin inhibited the activation of the AKT/GSK-3*β* pathway, thereby alleviating EMT mediated by TGF-*β*2.

### 3.7. Knockdown of Nrf2 Abolished the Inhibitions of Luteolin on H_2_O_2_-Induced EMT and Activation of the AKT/GSK-3*β* Pathway

**Figure 7:**
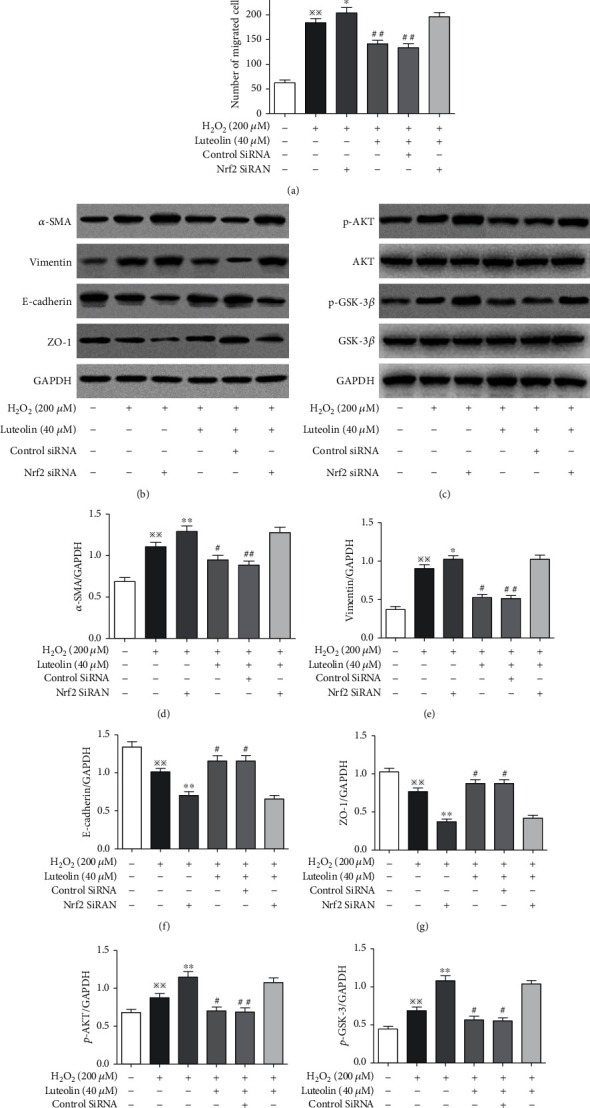
Knockdown of Nrf2 abolished the inhibitions of luteolin on H_2_O_2_-induced EMT and activation of the AKT/GSK-3*β* pathway. APRE-19 cells were treated with H_2_O_2_ and luteolin at 40 *μ*M. (a) Transwell migration for detecting the number of migrating cells. Data are expressed as mean ± SD (*n* = 3). Scale bar = 200 *μ*m. ^※※^*P* < 0.01 versus normal control; ^∗^*P* < 0.05 versus H_2_O_2_; ^##^*P* < 0.01 versus H_2_O_2_. Western blotting to evaluate the expression of *α*-SMA, vimentin, ZO-1, and E-cadherin (b, d–g) and AKT, p-AKT, GSK-3*β*, and p-GSK-3*β* (c, h, i). Data are expressed as mean ± SD (*n* = 3). ^※※^*P* < 0.01 versus normal control; ^∗^*P* < 0.05 and ^∗∗^*P* < 0.01 versus H_2_O_2_; ^##^*P* < 0.01 and ^#^*P* < 0.05 versus H_2_O_2_.

To investigate whether the protective effects of luteolin against H_2_O_2_-induced EMT was dependent on the Nrf2 pathway, ARPE-19 cells were transfected with siRNA specific to Nrf2. Transwell migration assays revealed that the cell migration number was increased in siNrf2-transfected cells compared with the H_2_O_2_ control cells. Compared to the H_2_O_2_ control cells, the migration number in luteolin-pretreated cells without siNrf2-transfection was significantly reduced, but not in siNrf2-transfected cells (Figure 7(a)). Western blot results showed that the expression of *α*-SMA and vimentin was significantly upregulated, and ZO-1 and E-cadherin were downregulated in siNrf2-transfected cells compared with the H_2_O_2_ control cells. Compared to the H_2_O_2_ control cells, the expression of *α*-SMA and vimentin was significantly downregulated, and ZO-1 and E-cadherin were upregulated in luteolin-pretreated cells without siNrf2 transfection, but not in siNrf2-transfected cells (Figures 7(b) and 7(d)–7(g)). To explore the possible association between the AKT/GSK-3*β* and Nrf2 pathway in H_2_O_2_-induced EMT, we examined the expressions of AKT, p-AKT, GSK-3*β*, and p-GSK-3*β*. The results showed that the expressions of p-AKT and p-GSK-3*β* were significantly increased in siNrf2-transfected cells compared with the H_2_O_2_ control cells. Compared to the H_2_O_2_ control cells, the expressions of p-AKT and p-GSK-3*β* were decreased in luteolin-pretreated cells without siNrf2-transfection, but not in siNrf2-transfected cells (Figures 7(c), 7(h), and 7(i)). Our results strongly suggested that inhibition of luteolin on H_2_O_2_-induced EMT in ARPE-19 cell through the AKT/GSK-3*β* pathway is dependent on the activation of the Nrf2 pathway.

## 4. Discussion

Clinically, AMD is divided into two types: dry and wet. The treatment of wet AMD, which accounts for about 15% of AMD, has yielded encouraging outcome with antivascular endothelial growth factor (VEGF) drugs. However, there are no available options for the treatment or prevention of dry AMD, which is the more common type, comprising 85% of all AMD cases [27]. Thus, intense efforts have been made to identify possible therapeutic targets to reduce disease progression. Luteolin is the main active ingredient of *Fructus Broussonetiae*, an herb commonly used for clinical treatment of age-related eye disease, including AMD. Therefore, luteolin was selected for the preliminary study on the prevention and treatment of AMD.

Oxidative damage from various sources, such as light exposure, inflammation, and oxidative stress, to the retina has been strongly linked with AMD [28, 29]. Therefore, an antioxidant could have therapeutic potential in the protection of RPE cells from oxidative damages and thus, it could be beneficial against AMD, at least against the dry form of the disease. The AREDS study suggested that antioxidant supplementation could have protective effects against AMD [30]. Many natural flavonoids show strong antioxidant activity and have the potential to treat dry AMD. Morin protected RPE cells from cigarette smoke extract through reduced oxidative stress, ER stress, and lipid accumulation via activated AMPK-Nrf2-HO-1 signaling pathway [31]. Our previous studies confirmed that quercetin and apigenin reduce oxidative damage in ARPE-19 cells and retina in model mice of dry AMD by activating the Nrf2 pathway [13, 24]. It also was reported that luteolin activates the Nrf2 pathway. For example, luteolin enhanced autophagy and antioxidative processes in both in vivo and in vitro models of intracerebral hemorrhage [32], protected heart tissues in STZ-induced diabetic mice through modulating Nrf2-mediated oxidative stress and NF-*κ*B-mediated inflammatory responses, and alleviates aflatoxin B_1_-induced apoptosis and oxidative stress in the liver of mice through activation of the Nrf2 pathway [33]. The current results showed that luteolin promoted nuclear translocation of Nrf2, upregulated the expressions of HO-1 and NQO-1, increased the activities of SOD and GSH-PX, and decreased intracellular levels of ROS and MDA, suggesting that luteolin activated the Nrf2 pathway, thereby improving the survival rate of oxidative damaged ARPE-19 cells. It is well documented that oxidative stress would also induce EMT [34–36]. However, available reports on EMT-related ophthalmic diseases have focused more on proliferative vitreoretinopathy than AMD [37, 38]. In the present study, we found that H_2_O_2_ significantly increased the number of cell migration, upregulated the expression of *α*-SMA and vimentin, and downregulated the expression of ZO-1 and E-cadherin, which was consistent with the EMT events in APRE-19 cells. Simultaneously, treatment of ARPE-19 cells with luteolin inhibited H_2_O_2_-induced cell migration and reversed the expression of epithelial markers ZO-1 and E-cadherin and mesenchymal markers *α*-SMA and vimentin. These results strongly suggested that the protective effect of luteolin on H_2_O_2_-induced EMT may be closely related to the activation of the Nrf2 pathway.

RPE is the main source of TGF-*β* in the outer retina. TGF-*β* can be activated in various ways such as heat, ultraviolet radiation, acidic pH, and ROS [39]. Once activated, TGF-*β* binds to specific transmembrane serine/threonine kinase receptors to transduce its intracellular signal by phosphorylating the canonical Smad pathway or noncanonical pathways, including the MAPK, PI3K/AKT, and mTOR that intricately modulate distinct downstream TGF-*β* responses [40]. It was reported that activation of the PI3K/AKT pathway leads to the phosphorylation and inactivation of GSK-3*β*, which triggers EMT [41]. In this study, we observed that the expression of TGF-*β*2 was upregulated in H_2_O_2_-treated ARPE-19 cells and downregulated in luteolin-treated cells. Correspondingly, the expressions of p-AKT and p-GSK-3*β* were increased in H_2_O_2_-treated ARPE-19 cells and decreased in luteolin-treated cells. These data indicated that luteolin inhibited the activation of the AKT/GSK-3*β* pathway.

Studies have supported that there is a strong relationship between the Nrf2 pathway and AKT/GSK-3*β* pathway in the process of EMT. Melatonin inhibits LPS-induced EMT in human alveolar epithelial cells depending on upregulation of the Nrf2 pathway mediated by the PI3K/GSK-3*β* axis [42]. In renal interstitial fibrosis of unilateral ureteral obstruction mice, the antifibrotic effect of Nrf2 is imparted by the inactivation of PI3K/Akt signaling [15]. In this study, we found that cell migration number was increased and the expression of *α*-SMA and vimentin was upregulated, and ZO-1 and E-cadherin were downregulated in siNrf2-transfected cells than in siNrf2-untransfected cells. Luteolin could inhibit cell migration and reversed the expressions the EMT-related proteins in siNrf2-untransfected cells but had no effects in siNrf2-transfected cells. Further, we also found that the expressions of p-AKT and p-GSK-3*β* were increased in siNrf2-transfected cells than in siNrf2-untransfected cells and decreased in luteolin-pretreated siNrf2-untransfected cells but did not in siNrf2-transfected cells. These results further illustrate that the inhibition of luteolin on EMT in H_2_O_2_-treated ARPE-19 cells through the AKT/GSK-3*β* pathway is dependent on the activation of the Nrf2 pathway.

In conclusion, in this study, we simultaneously studied the effects of oxidative stress on the Nrf2 pathway and EMT and discussed the relationship between two signaling pathways and the influence of luteolin intervention. The results showed that the inhibition of luteolin on EMT induced by oxidative injury in ARPE-19 cell through the AKT/GSK-3*β* pathway is dependent on the activation of the Nrf2 pathway. Our current study indicated that luteolin could be a potential drug for the treatment of dry AMD. However, luteolin has the disadvantages of poor water and fat solubility and low bioavailability. Further research is needed to change its dosage forms, such as preparation of phospholipid complex and solid dispersion; to improve its bioavailability; and then to verify its effect on dry AMD through in vivo and in vitro experiments. It may be expected that luteolin will have a good application prospect in the treatment of dry AMD after solving the problem of low biological activity.

## Data Availability

The data presented in this study are available on request from the corresponding author.

## Abbreviations

AKT: Protein kinase B
AMD: Age-related macular degeneration
EMT: Epithelial-mesenchymal transition
GSH-Px: Glutathione peroxidase
GSK-3*β*: Glycogen synthase kinase-3beta
HO-1: Heme oxygenase-1
MDA: Malondialdehyde
NQO1: NAD(P)H quinone dehydrogenase 1
Nrf2: Nuclear factor E2-related factor 2
ROS: Reactive oxygen species
RPE: Retinal pigment epithelium
*α*-SMA: Alpha-smooth muscle actin
SOD: Superoxide dismutase
TGF-*β*2: Transforming growth factor-beta 2
ZO-1: Zonula occludens 1.
